# Development of the Functionalized Nanocomposite Materials for Adsorption/Decontamination of Radioactive Pollutants

**DOI:** 10.3390/ma14112896

**Published:** 2021-05-28

**Authors:** Gyo Eun Gu, Joonwon Bae, Ho Seok Park, Jin-Yong Hong

**Affiliations:** 1 Center for C1 Gas & Carbon Convergent Research, Korea Research Institute of Chemical Technology (KRICT), 141 Gajeong-ro, Yuseong-gu, Daejeon 34114, Korea; bodk7447@gmail.com; 2 School of Chemical Engineering, Sungkyunkwan University (SKKU), Suwon 16419, Korea; 3 Department of Applied Chemistry, Dongduk Women’s University, Seoul 02748, Korea; redsox7@dongduk.ac.kr; 4 Advanced Materials and Chemical Engineering, University of Science and Technology (UST), Daejeon 34113, Korea

**Keywords:** Prussian blue, electrospinning, radionuclide, adsorption, decontamination

## Abstract

A polymer-based nanofiber membrane with a high specific surface area, high porosity and abundant adsorption sites is demonstrated for selective trapping of radionuclides. The Prussian blue (PB)/poly(methyl methacrylate) (PMMA) nanofiber composites were successfully prepared through a one-step, single-nozzle electrospinning method. Various analytical techniques were used to examine the physical and chemical properties of PB nanoparticles and electrospun nanofibers. It is possible to enhance binding affinity and selectivity to radionuclide targets by incorporation of the PB nanoparticles into the polymer matrix. It is noteworthy that the maximum ^133^Cs adsorption capacity of hte PB/PMMA nanofiber filter is approximately 28 times higher than that of bulk PB, and the removal efficiency is measured to be 95% at 1 ppm of ^133^Cs. In addition, adsorption kinetics shows that the PB/PMMA nanofiber has a homogenous surface for adsorption, and all sites on the surface have equal adsorption energies in terms of ion-exchange between cyano groups of the introduced PB nanoparticles and radionuclides.

## 1. Introduction

Nuclear power plants have generated large quantities of highly radioactive materials due to the deterioration of nuclear facilities and unpredictable accidents [1,2]. This fact has aroused serious questions regarding the substantial danger of nuclear power plants, which has been overlooked for a long time. Concerns about wastewater treatment from nuclear plants are also increasing. For example, enormous amounts of radioisotopes have been spilled over into the soil and ocean, etc. Consequently, a policy that pursues reduction in nuclear power plants has been declared recently in many countries including Germany, Italy, and Japan. As most nuclear power plants become old and outdated, levels of radiations from nuclear wastes become higher. Therefore, extreme care must be provided to protect people and facilities while handling radioactive materials and operating the facilities. It is important to develop a technology for the reduction in nuclear waste along with an automation system to minimize exposure from radioactivity [3,4,5,6].

Among the radioactive contaminants, elemental species such as caesium (^137^Cs), strontium (^90^Sr) and cobalt (^60^Co) can continuously damage both human life and environment even by exposure with a trace amount [7,8]. The most harmful radionuclides, ^137^Cs, has a long half-life of 30.2 years, high solubility, and strong gamma-ray emission. Accordingly, it should be removed completely for decontamination and protection of human life [9,10,11,12,13].

Even if some candidates might be present, ferric hexacyanoferrate (Prussian blue; PB) is interesting because it is known as a selective adsorbent for alkali cations, which is evidenced by the earlier studies [14,15]. PB is a zeolite-like inorganic coordination compound in which a high spin Fe^3+^ and a low spin Fe^2+^ was surrounded by six nitrogen and carbon atoms of cyanide groups on a face centered cubic lattice [16,17]. The adsorption mechanism between PB particles and caesium ions can be mainly explained with two probable reasons: (1) The crystal lattice length of PB (5.1 Å) is sufficiently longer than the hydration radii of the Cs^+^ cations (3.25 Å) and (2) PB can exchange its potassium ions with caesium ions [18,19,20,21]. In addition, PB has been considered as the most appropriate material for selective trapping of the radioactive caesium ions thanks to its low cost and facile synthesis compared with other adsorbents. Prussian blue has become even more popular since the Fukushima nuclear accident [22,23,24,25].

It is still challenging to develop a new technology that can prevent the spread of radioactive contaminants from nuclear power plants. To date, there have been studies on adsorbent materials using the PB nanoparticles for control over the radioactive caesium. Some research groups have reported techniques with PB nanoparticles for the reduction of contaminated radionuclides [26,27]. However, the fundamental difficulty in getting rid of pollutants still exists. Moreover, research into materials and systems that can adsorb and eliminate radionuclides has been scarcely performed yet.

Herein, a new web-type composite filter that can effectively adsorb radioactive contaminants was fabricated by using the electrospinning method. The filter was produced by a synergistic combination of polymeric nanofibers and incorporated PB nanoparticles. A synergy effect is anticipated by combining the high specific surface area of the nanofibers with PB nanoparticles possessing an excellent adsorption performance. Electrospinning is a method of producing continuous fibers by applying an electric filed to a polymer solution at a high voltage. It is easy to control nanofiber diameter, increase surface area, and compounds with other inorganic nanomaterials [28,29]. Poly(methyl methacrylate)(PMMA), one of the representative thermoplastic polymers, widely referred to as “safety glass” and has been selected as a nanofiber matrix, because fibrous PMMA is significantly resistant to most environmental parameters and its properties remain intact after electrospinning [30,31,32]. Meanwhile, it is necessary to immobilize strongly the PB nanoparticles to the polymer matrix, because the dispersion of PB nanoparticles in water might cause secondary contamination. It was possible to encapsulate the PB nanoparticles in the PMMA matrix and re-dispersion could be suppressed by the electrospinning method. The obtained PB/PMMA filter was analyzed with diverse instrumentations. The adsorption behavior of radionuclides to the PB/PMMA filter was systematically investigated as a function of parameters such as contact times, initial concentration, type of radionuclides, and pH. Additionally, the feasibility of filter reuse was also demonstrated in the recycling system.

## 2. Materials and Methods

### 2.1. Materials

Chemicals such as *N*, *N*-dimethylformamide (DMF, >99.8%), poly(methyl methacrylate) (PMMA, average molecular weight of 350,000 g/mol), iron(III) chloride (FeCl_3_, 97%), potassium hexacyanoferrate(III) (K_3_Fe(CN)_6_, ≥99%), caesium standard for ICP and barium, strontium, and rubidium standard for ICP were purchased from Sigma Aldrich Chemical Co. (Milwaukee, WI, USA). Ammonia solutions and hydrochloric acid (HCl) were purchased from Samchun Chemical (Seoul, Korea). All chemicals were used as received without further purification.

### 2.2. Fabrication of Prussian Blue Nanoparticles

The PB nanoparticles were prepared by a chemical precipitation method. Typically, FeCl_3_ (1.96 g, 0.012 mol) and K_3_[Fe(CN)_6_] (0.64 g, 0.002 mol) were added to 100 mL distilled water at 55 °C and stirred at 300 rpm for 12 h. The precipitated Fe^III^_4_[Fe^II^(CN)_6_]_3_ nanoparticles were centrifuged, washed several times with deionized water, and then dried in an oven at 120 °C overnight.

### 2.3. Preparation of PB-Incorporated PMMA Nanofiber Composites Using Elecztrospinning

A precursor solution was prepared by dissolving 10 wt% of PMMA in DMF through vigorous stirring at 60 °C for 8 h at 300 rpm. After stirring, PB nanoparticles with different weight fractions (0%, 10%, 50%, 100% of PMMA weight) were added to the PMMA solution in DMF. Next, the solutions were sonicated for 90 min. After dispersion, the solutions were cooled down to room temperature. Subsequently, the homogeneous precursor solution was supplied by a disposable syringe that was connected to the electrospinning machine. An electric field of 10 kV was applied between the needle gap and a flat collector covered with aluminum foil with a fixed collection distance of 15 cm. The feed rate of the solution was controlled by syringe pump at 10 μL/min. During the electrospinning process, the temperature and humidity values were kept constant at 20 °C and 35%, respectively. The as-spun products were collected for 6 h and dried at 80 °C under vacuum overnight prior to further characterization.

### 2.4. Characterization

The morphology was confirmed using a Field Emission Scanning Electron Microscopy (FE-SEM) at 10 kV (Tescan Mira-3 FEG, Brno-Kohoutovice, Czech Republic). Fourier-transform infrared spectroscopy (FT-IR) spectra were recorded on Alpha-P (Bruker, Karlsruhe, Germany). X-ray diffraction (XRD) patterns were collected using a Ultima IV with Cu Kα radiation (Rigaku, Tokyo, Japan). The Thermogravimetric analysis (TGA) curves were performed from 25 to 1000 °C using a heating rate of 10 °C/min under N_2_ atmosphere (SDT Q600, TA Instruments, New Castle, DE, USA). The Brunauer–Emmett–Teller (BET) analyses were performed with an ASAP 2020 (Micromeritics, Norcross, GA, USA) using the N_2_ adsorption-desorption isotherms. The solutions were electrospun at room temperature using an electrospinning setup (ESR100D, NanoNC Co., Seoul, Korea). Inductively coupled plasma mass spectrometry (ICP-MS) was used to analyze and confirm the caesium concentration in solution (iCAP RQ, Thermo Fisher Scientific, Waltham, MA, USA).

### 2.5. Radioactive Material Adsorption Experiment

All adsorption experiments were performed using 250 mL tall Pyrex glass beaker in order to avoid undesired side reactions. The caesium, rubidium, barium, strontium, and cerium standard solution (1 ppm) containing 60% HNO_3_ solution was added to 100 mL of distilled water, respectively. All reagents were selected as representative radionuclides, and used in the form of the stable isotope. To control the pH, ammonia or hydrochloric acid solution was injected into the aqueous solutions. Then, 0.1 g of adsorbents (both PB@PMMA fabric filter and bulk PB) were introduced into the above solutions and shaken at 300 rpm according to the conditions, which would be suitable for ^133^Cs ion adsorption. The adsorption capacity for ^133^Cs ion was monitored from 0 min to 72 h. After the adsorption process, the solution was filtered through a syringe filter (PTFE-H, pore size 0.2 µm, Hyundai Micro) and then the inductively coupled plasma-Mass Spectrometer (ICP-MS) test was performed to determine the ^133^Cs ion concentration. To obtain accurate results, the ^133^Cs adsorption test was repeated three times and the averaged value (error range: ±5%) was provided.

## 3. Results and Discussion

### 3.1. Development of 3D Porous Filter Fabric with a Controlled Pore Structure and Physical Properties

The successful formation of PB nanoparticles is one of the most important prerequisites for this study. The amorphous PB nanoparticles could be spontaneously formed via a chemical reaction in aqueous solutions. For this coordination compound, adsorption of radioactive radionuclides was achievable by several specific interactions such physical adsorption, chemical ion-exchange, and interaction with bound water.

**Figure 1:**
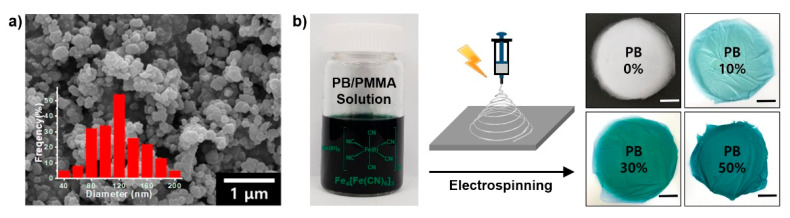
(**a**) SEM image of synthesized PB nanoparticles. The average nanoparticle size was determined by SEM (100 particles counted) and was illustrated in the histogram. (**b**) Schematic illustration for fabricating the PB/PMMA nanofiber composites. Photograph showing the electrospun nanofiber membranes with different concentrations of PB nanoparticles (scale bar: 3 cm).

To verify the PB nanoparticle formation, a scanning electron microscopy (SEM) image is shown in Figure 1a. Note that fairly spherical nanoparticles were effectively prepared by a simple reaction. The inset histogram observed by counting 200 particles indicated that the most frequently observed diameter was 120 nm and the size range was 40–200 nm, with a relatively broad size distribution. The most important parameter for the formation of PB nanoparticles is the molar ratio between ferric chloride and potassium ferricyanide, and the best value was 6:1 for this experiment. Subsequently, the resulting PB nanoparticles was mixed with PMMA as a solution and the solution was electrospun for 1 h to produce PB/PMMA nanofiber membrane filters as a function of PB concentration (Figure 1b). While the membranes containing the PB nanoparticles apparently showed a dark green color regardless of PB composition, the membrane color became white without PB nanoparticles.

**Figure 2:**
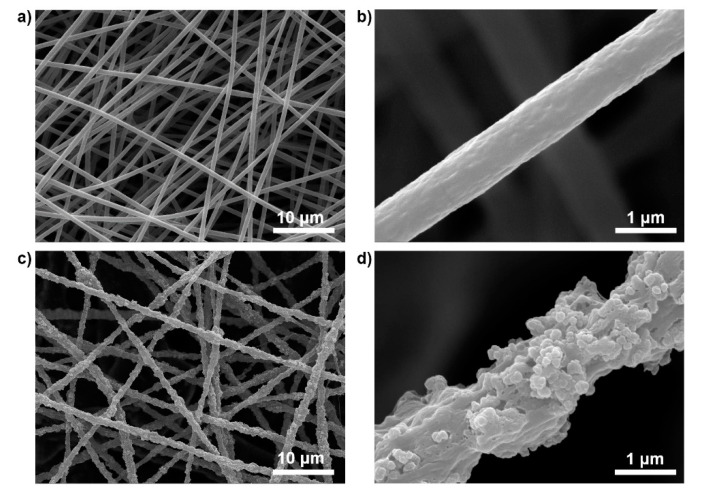
SEM images of (**a**,**b**) neat PMMA nanofibers prepared using electrospinning and (**c**,**d**) PB(50 wt%)/PMMA nanofibers at different magnifications.

The morphological feature of the PB/PMMA nanofiber filter was examined by SEM observation compared with that of PMMA nanofiber. Figure 2a shows that the electrospun PMMA nanofibers have a high aspect ratio and a flat surface with an average diameter of 800 ± 60 nm (50 randomly selected). The magnified image in Figure 2b also shows that traces of the PB nanoparticles are absent macroscopically. On the contrary, the surfaces of PB/PMMA nanofibers became rough and traces of nanoparticles were observed prevalently in contrast to the PMMA nanofibers (Figure 2c). A closer observation confirmed the presence of the incorporated PB nanoparticles in Figure 2d.

**Figure 3:**
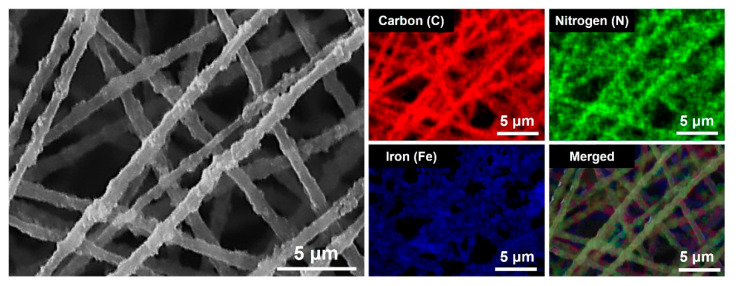
SEM image of the PB (50 wt%)/PMMA nanofibers, EDS mapping results for carbon (C), nitrogen (N) and Iron (Fe) elements, and their merged image (C + N + Fe). The EDS analysis was performed on the selected area of the electrospun nanofiber surface.

The elemental mapping of C, N and Fe obtained by EDS verified the successful addition of the PB nanoparticles to the PMMA matrix (Figure 3). Note that the signals of C, N and Fe atoms were co-localized the PB/PMMA nanofibers as shown in the overlay image. These findings indicate successful introduction of the PB nanoparticles onto the overall surface of PMMA nanofibers.

**Figure 4:**
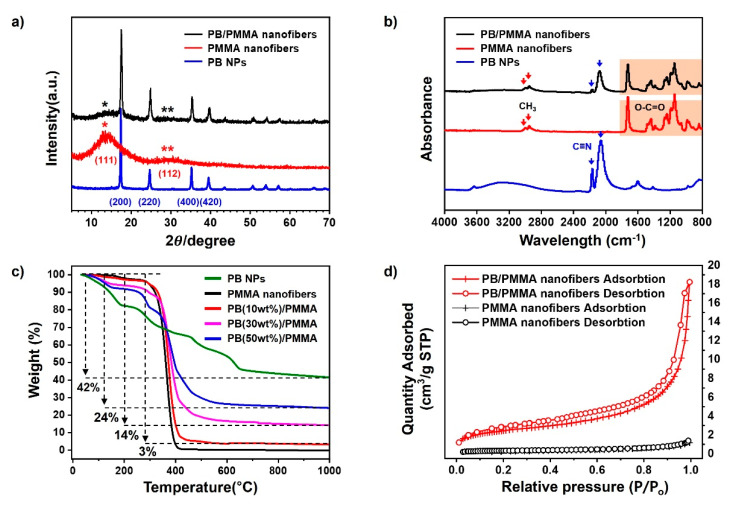
(**a**) X-ray diffraction analysis; (**b**) FT-IR spectroscopy; (**c**) Thermogravimetric analysis; and (**d**) Nitrogen adsorption/desorption isotherms analysis of the PB nanoparticles, PMMA nanofibers and PB/PMMA nanofibers, respectively. The PB contents of PB/PMMA nanofiber samples in (**a**,**b**,**d**) is 50 wt%.

The additional physical and chemical properties of the PB nanoparticles, PMMA and PB/PMMA nanofiber filter such as crystallinity, thermal stability, and nitrogen adsorption/desorption, were examined using diverse instrumentations. The XRD pattern of PMMA nanofibers (red line) in Figure 4a shows two broad peaks at the vicinity of 14.0° and 30.7° associated with an amorphous structure [33]. It is difficult to observe the highly crystalline character from the XRD profile, as a sharp secondary peak is undiscernible. On the other hand, the XRD profile of PB nanoparticles (blue line) showed a significantly different trend. The representative primary and secondary peaks were observed at around 17 and 34 degrees, respectively. Several additional peaks also appeared due to highly crystalline face-centered cubic (FCC) phase. Sharp diffraction peaks at 17.5°, 24.8°, 35.3°, and 39.5° were attributed to PB(200), PB(220), PB(400), and PB(420), respectively [34]. These lattice parameters are in good agreement with the standard data of PB (ICDD PDF2 01-073-0687), and the half-width at 2*θ* = 17.5° enables us to determine 70 nm of the crystallite size by using Scherrer’s Equation. These features were also observed in the XRD pattern of the PB/PMMA nanofibers (black line). The blue and black lines were overlapped significantly, supporting the presence of the PB nanoparticles in the PMMA matrix.

FT-IR spectra are presented in Figure 4b. The PMMA spectrum clearly shows the typical peaks at around 3000, 1700, 1390, and 1250 cm^−1^ due to C-H stretching, carbonyl stretching, α-methyl vibrations, and C-O bending, respectively. It was remarkable that the cyanide stretching peak at 2050 cm^−1^ assigned as the C≡N stretching vibration group in potassium hexaferricyanide was observed in the PB nanoparticle spectrum. All these characteristic peaks were observed in the spectrum of the PB/PMMA nanofiber filter. The splitting of the CN band at 2050 cm^−1^ could be explained following two possible reasons.

The first was the transition of Prussian Blue to Berlin Green. As the solution containing iron(III) chloride and potassium hexacyanoferrate(III) was unstable, Prussian Blue oxidized to Prussian Yellow, which easily degraded in the solution state. This Prussian Yellow partially oxidized and decomposed to Berlin green. As the Berlin green possessed a mixed state, it was split into two peaks. In addition, the occurrence of the second CN peak at 2160 cm^−1^ was associated with the chelation formation between PB nanoparticles. Weaker bands at 3300 and 1600 cm^−1^ were associated with the O–H stretch and H–O–H bending mode from interstitial water, which disappeared in the spectrum of the PB/PMMA nanofiber filter [35].

The substantial compositions of the PB nanoparticles in the PB/PMMA nanofiber filters were determined using thermogravimetric analysis (TGA) and the results were summarized in Figure 4c. PB nanoparticles and PB/PMMA nanofiber filters with varying PB compositions were heated from room temperature to a fixed temperature. The thermogram of the PB nanoparticles was unique, showing 4 major stepwise degradations. Distinctive categorization was very difficult, the first (below 200 °C) and third (450–650 °C) weight loss was due to loss of residual moisture and decomposition of cyanide groups [36]. The TGA profile of the PMMA nanofiber showed two major decompositions. The first one below 100 °C was associated with the removal of low molecular weight compounds and the second major decomposition at around 300 °C was attributed to PMMA degradation. When the PB nanoparticles and PMMA nanofibers were pyrolyzed completely, the resulting weight fractions were 42 and 3%, respectively (green and black line). This meant that the organic PMMA component was completely removed by heating. Considering this value, the char yields from the PB/PMMA nanofibers, 3, 14 and 24%, were reasonable. This was indirect evidence confirming the incorporation of PB nanoparticles to PMMA matrix.

Figure 4d shows nitrogen adsorption/desorption isotherms of the PMMA and PB/PMMA nanofiber filter. The specific surface area of the PMMA and PB/PMMA nanofibers were 1.25 and 9.19 m^2^/g, respectively. The addition of PB nanoparticles onto PMMA fibers increased the surface area and pore volume. This increase was critical for performance improvement of PB/PMMA nanofiber filters by promoting caesium adsorption.

### 3.2. Adsorption/Decontamination Performances of the PB/PMMA Nanofiber Filters

**Figure 5:**
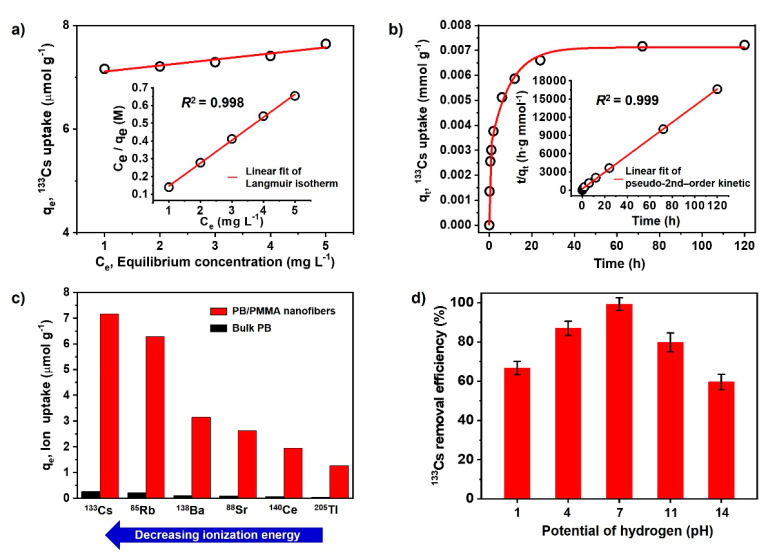
(**a**) Adsorption isotherms of ^133^Cs onto PB/PMMA nanofibers by equilibrium concentration (inset: Langmuir isotherm); (**b**) Adsorption of ^133^Cs onto PB/PMMA nanofibers by the contact time (inset: pseudo-second order kinetics); (**c**) The elimination capacity of PB/PMMA nanofibers to the 6 radionuclides; (**d**) pH effect on the ^133^Cs uptake onto the PB/PMMA nanofibers at 20.0 °C. The PB contents of PB/PMMA nanofiber sample is 50 wt%.

To investigate the adsorption (uptake) capacity and removal efficiency of the PB/PMMA nanofiber filters, stable ^133^Cs was used instead of radioactive ^137^Cs. Figure 5a shows the ^133^Cs ion uptake by the PB/PMMA nanofiber filters as a function of the equilibrium ^133^Cs concentration at 20 °C and pH 7.0. In this study, the adsorption equilibrium was gradually established for 72 h. Accordingly, ^133^Cs adsorption capacity of solutions was measured for 72 h contact time. The ^133^Cs uptake was calculated by the following equation:

(1) $$q_{e}=\frac{\left(C_{o}-C_{e}\right)V}{AW}$$

where $q_{e}$ is the equilibrium adsorption capacity of the adsorbent in mmol/g, $C_{o}$ the initial concentration of the ^133^Cs in mg L^−1^, $C_{e}$ the equilibrium concentration of ^133^Cs after adsorption in mg L^−1^, *V* the total volume of solution in L, and *A* the atomic weight of ^133^Cs in g/mol, and *W* the weight of the PB/PMMA microfibers in g, respectively.

To gain some insight regarding the ^133^Cs adsorption process by the PB/PMMA nanofibers, the equilibrium uptake (adsorption) data were fitted to Langmuir and Freundlich isotherms.

The Langmuir adsorption isotherm describes that the surface is homogeneous assuming that all the adsorption sites at surface have an identical adsorbate affinity and that adsorption at one site does not influence adsorption at adjacent sites [37]. Furthermore, each adsorbate molecule must be located on a single site and a monolayer is formed onto the adsorbent surface. The Langmuir Equations are as follows:

(2) $$q_{e}=\frac{q_{m}bC_{e}}{1+bC_{e}}\;\left(\mathrm{nonlinear}\;\mathrm{form}\right)\;\mathrm{or}\;\frac{C_{e}}{q_{e}}=\left(\frac{1}{q_{m}b}\right)+\left(\frac{1}{q_{m}}\right)C_{e}\;\left(\mathrm{linear}\;\mathrm{form}\right)$$

where $q_{m}$ is the maximum 133Cs uptake amount in mmol/g, b is a constant that refers to the bonding energy of adsorption correlated to free energy and net enthalpy in L/mg. On the contrary, the Freundlich model is based on the reversible adsorption on the heterogeneous surface since it does not restrict the monolayer formation [38].

A Freundlich adsorption isotherm can be defined by the following Equation:

(3) $$q_{e}=K_{f}C_{e}^{1/n}\;\left(\mathrm{nonlinear}\;\mathrm{form}\right)\;\mathrm{or}\;\log\;q_{e}=\log\;K_{f}+\frac{1}{n}\log\;C_{e}\;\left(\mathrm{linear}\;\mathrm{form}\right)$$

where $K_{f}$ is a constant related to the adsorption capacity of the adsorbent in mmol/g, and *1/n* is the intensity of the adsorption constant. Even if the $q_{m}$ and $K_{f}$ are fundamentally different, the same conclusion is reached. It is known that $q_{m}$ is the monolayer adsorption capacity, while $K_{f}$ is the relative adsorption capacity. Nonlinear regression analysis was carried out by computer software in order to evaluate the parameters. The $q_{m}$, *b*, $K_{f}$, *n* values and the nonlinear regression correlations for Langmuir (*R*^2^), Freundlich (*R*^2^) isotherms are listed in Table 1.

Based on these data, it showed that the Langmuir isotherm fitted better with regression coefficient R^2^ = 0.998 and acceptable parameter errors. In particular, the Langmuir isotherm can be represented in terms of a dimensionless constant separation factor (*R_L_*) [39]. The *R_L_* is equal to the ratio of the unused adsorbent capacity to the maximum adsorbent capacity. It can be considered as an affinity between the adsorbate and adsorbent. The *R_L_* value is calculated by the following Equation:

(4) $$R_{L}=\frac{1}{1+bC_{0}}$$

where *b* and $C_{0}$ are the Langmuir constant and initial concentration of ^133^Cs. In general, *R_L_* is classified as *R_L_* > 1, *R_L_* = 1, 0 < *R_L_* < 1 and *R_L_* = 0, indicating that the type of adsorption isotherm is unfavorable, linear, favorable and irreversible, respectively. The obtained *R_L_* value was approximately 0.117, suggesting that the adsorption of ^133^Cs on the PB/PMMA nanofibers was favorable. Accordingly, the as-synthesized PB/PMMA nanofibers have a homogenous surface for ^133^Cs ion adsorption and all sites have an equal adsorption energy.

Figure 5b shows ^133^Cs uptake by the PB/PMMA nanofibers as a function of contact time. It indicates that the adsorption capacity was saturated after 72 h. To understand the ^133^Cs ion adsorption kinetics to the PB/PMMA nanofibers, the experimental data were fitted to the pseudo-first-order [40] and pseudo-second-order kinetic [41] model (Insets in 5b). To analyze the ^133^Cs ion uptake rates as a function of time, the pseudo-first-order kinetic equation is used:

(5) $$\log\left(q_{e}-q_{t}\right)=\log q_{e}\;-\;\frac{K_{1}}{2.303}t$$

where $q_{e}$ and $q_{t}$ are the ^133^Cs uptake at equilibrium and time *t*, respectively, and *K_1_* the constant of first-order adsorption in min^−1^. The pseudo-second-order equation is given as:

(6) $$\frac{t}{q_{t}}=\frac{1}{K_{2}q_{e}{}^{2}}+\frac{t}{q_{e}}$$

where, *K_2_* is the rate constant of second-order adsorption in g/mmol·min. Considering the results in Table 1, the adsorption behavior as a function of time could be explained effectively by a pseudo-second-order model with the regression coefficient *R^2^* = 0.999 and acceptable errors for parameters. In general, the pseudo-second-order kinetic model assumes that the adsorption process occurs on localized sites without interactions between adsorbates and maximum adsorption is achieved when the adsorbate monolayer is generated on the adsorbent surface. The desorption rate is negligible compared to the adsorption rate. The chemical interactions induced by a sharing or exchange of electrons between C≡N groups of the introduced PB nanoparticles and ^133^Cs ions can be one of the major driving forces.

It was expected that the PB/PMMA nanofibers can uptake other various radionuclides such as ^85^Rb, ^88^Sr, and ^138^Ba, in case their size is similar to that of ^133^Cs. Therefore, the uptake performance of the PB/PMMA nanofiber filters to the six different radionuclides was measured at pH 7.0 and 20 °C compared with bulk PB (control group). As shown in Figure 5c, it was apparent that the performance of the PB/PMMA was much higher than that of bulk PB. It is noteworthy that the maximum ^133^Cs adsorption capacity of the PB/PMMA nanofiber filter is approximately 28 times higher than that of bulk PB. This is attributed to the high surface area and PB contents in the synthesized PB/PMMA nanofibers, providing abundant active sites for ^133^Cs adsorption. The maximum uptake values of PB/PMMA nanofiber filters were evaluated as approximately 7.16 (^133^Cs), 6.28 (^85^Rb), 3.14 (^138^Ba), 2.61(^88^Sr), 1.95(^140^Ce) and 1.26 (^205^Tl) μmol/g, respectively. In particular, it is notable that the adsorption capacity increased with the decreasing ionization energy and electronegativity of the radioactive species. Under this experimental condition, caesium has the lowest ionization energy (1st ionization energy: 375.7 kJ/mol) and the lowest electronegativity (0.79), resulting in a high adsorption capacity.

The dependence of ^133^Cs removal efficiency on pH was tested and presented in Figure 5d. The removal efficiency increased in acidic conditions and decreased in basic media. It showed a maximum removal efficiency closely associated with the adsorption capacity at pH 7.0. This was probable because the ^133^Cs ion formation efficiency was influenced by the pH of the media, because ionization was a charge-controlled process.

### 3.3. Replaceable Performances of the PB/PMMA Nanofiber Filters

**Figure 6:**
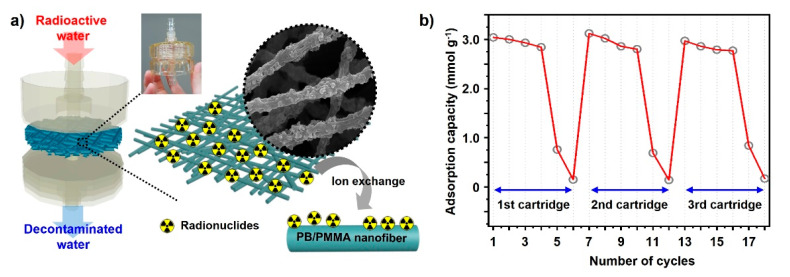
(**a**) A schematic representation of the PB/PMMA nanofiber membrane introduced in the form of a cartridge. The corresponding images showing the radionuclides adsorption mechanism of PB/PMMA nanofibers. (**b**) The radionuclides adsorption cycle test of the electrospun PB (50 wt%)/PMMA nanofiber membranes. The cartridge was replaced after 6 times performing each test.

The water treatment process by eliminating caesium ions from contaminated water is simply illustrated in Figure 6a. The circulation system is propelled by the main driving force, constant pressure provided by the pump. As the cycling system operates, the amount of radioactive caesium ions in the water bath decreases drastically. PB-incorporated PMMA non-woven fabric is sandwiched between water tanks as a water treatment filter.

During system operation, the caesium ions are trapped inside the PB nanoparticles in the PB/PMMA filter. The potassium ions embedded in the PB are exchanged with radiative caesium ions during the purification. To demonstrate the caesium removal capability of the filter fabric, caesium concentration was precisely measured using ICP-Mass. Differences in caesium concentrations were calculated with the ICP-Mass results before and after the uptake experiments under various conditions in solution.

The cycle test result is shown in Figure 6b, indicating that the removal efficiency of the PB/PMMA nanofiber filter was maintained even after the fourth cycle. However, it decreased dramatically during the fifth cycle and the capacity deteriorated almost completely after the sixth cycle. This trend was observed repeatedly after cartridge replacement. We can easily determine accurate points for cartridge substitution. In this study, 0.1 g of the PB-incorporated PMMA filters were employed, because this amount was generally sufficient to perform uptake tests, considering the caesium concentration released after the Fukushima accident.

## 4. Conclusions

In this work, the feasibility of the most common radionuclides, caesium ion removal using electrospun PB/PMMA nanofiber filters was demonstrated experimentally, as PB is considered suitable and effective for caesium removal. Because direct dispersion of PB nanoparticles in water can cause a secondary contamination, incorporation of the PB nanoparticles in the PMMA matrix was employed by electrospinning in this work. The successful formation of the PB/PMMA nanofiber filter membrane was substantially confirmed by diverse instumentations such as FT-IR, XRD, SEM including EDX, TGA and BET. The results supported that the PB nanoparticles were successfully introduced into the PMMA matrix without changing their intrinsic crystallinity and structure. The PB/PMMA nanofiber filter showed the maximum adsorption(uptake) capacity 7.3 µmol/g and the high removal efficiency over 95% in 1 ppm of caesium ion solution. The adsorption mechanism was measured experimentally and analyzed numerically using two typical isotherms. It was found to follow Langmuir isotherm and the pseudo-2nd-order kinetic mechanism. The PB/PMMA nanofiber filter showed the best removal capacity to Cs compared to other radionuclides and the removal efficiency value reached a maximum at pH 7.0. The replaceability of the PB/PMMA nanofiber filter was demonstrated under continuous purification conditions, showing that the uptake capacity was approximately 3.0 µmol/g and the capacity was maintained after the fourth cycle. Consequently, it was concluded that the PB/PMMA nanofiber filter could act as a “chemical sponge” (high-performance adsorbent) for simple decontamination at a reduced cost. This study can provide important information for future research activities regarding water purification technology through nuclear waste removal, because the amount of radionuclides released increases explosively due to nuclear power generation.

## Figures and Tables

**Table 1:** Adsorption parameters of the Langmuir, Freundlich, pseudo-first-order and pseudo-second-order models at room temperature for the adsorption of ^133^Cs ion on PB/PMMA nanofibers.

	Langmuir			Freundlich			Pseudo 1st Order			Pseudo 2nd Order		
	*q_m_* _[a]_ (µmol/g)	*b* (L/mg)	*R* ^2 [b]^	*K_f_* (µmol/g)	*n*	*R* ^2 [b]^	*K_1_* (min^−1^)	*q_e1_* (µmol/g)	*R* ^2 [b]^	*K_2_* (min^−1^)	*q_e2_* (µmol/g)	*R* ^2 [b]^
^133^Cs	7.75	7.55	0.99	7.09	27.92	0.68	0.005	162.6	0.84	87.74	0.01	0.99

^[a]^ The *q_m_*, *b*, *K_f_*, *n*, *K_1_*, *K_2_*, *q_e1_*, *q_e2_* values and the nonlinear regression correlations for Langmuir, Freundlich isotherms, pseudo-first-order and pseudo-second-order models were measured by nonlinear regression analysis using OriginPro 8.5. ^[b]^ *R*^2^ = regression coefficient.

## Data Availability

Data sharing is not applicable to this article.

## References

[1] Gavrilescu M., Pavel L.V., Cretescu I. (2009). Characterization and remediation of soils contaminated with uranium. J. Hazard. Mater..

[2] Talan D., Huang Q. (2021). Separation of radionuclides from a rare earth-containing solution by zeolite Adsorption. Minerals.

[3] Boulanger N., Kuzenkova A.S., Iakunkov A., Romanchuk A.Y., Trigub A.V., Egorov A.V., Bauters S., Amidani L., Retegan M., Kvashnina K.O. (2020). Enhanced sorption of radionuclides by defect-rich graphene oxide. ACS Appl. Mater. Interfaces.

[4] Lyczko M., Wiaderek B., Bilewicz A. (2020). Separation of radionuclides from spent decontamination fluids via adsorption onto titanium dioxide nanotubes after photocatalytic degradation. Nanomaterials.

[5] Obaid S.S., Gaikwad D.K., Sayyed M.I., Al-Rashdi K., Pawar P.P. (2018). Heavy metal ions removal from waste water by the natural zeolites. Mater. Today.

[6] El-Deen S.E.A.S., El-Deen G.E.S., Jamil T.S. (2019). Sorption behavior of co-radionuclides from radioactive waste solution on graphene enhanced by immobilized sugarcane and carboxy methyl cellulose. Radiochim. Acta.

[7] Skwarek E., Janusz W. (2019). Adsorption of Ba^2+^ ions at the hydroxyapatite/NaCl solution interface. Adsoption.

[8] Janusz W., Skwarek E. (2016). Study of sorption processes of strontium on the synthetic hydroxyapatite. Adsoption.

[9] Olatunji M.A., Khandaker M.U., Mahmud H.E., Amin Y.M. (2015). Influence of adsorption parameters on caesium uptake from aqueous solutions-a brief review. RSC Adv..

[10] Majidnia Z., Idris A. (2015). Evaluation of caesium removal from radioactive waste water using maghemite PVA–alginate beads. Chem. Eng. J..

[11] Sangvanich T., Sukwarotwat V., Wiacek R.J., Grudzien R.M., Fryxell G.E., Addleman R.S., Timchalk C., Yantasee W. (2010). Selective capture of caesium and thallium from natural waters and simulated wastes with copper ferrocyanide functionalized mesoporous silica. J. Hazard Mater..

[12] Ofomaja A.E., Pholosi A., Naidoo E.B. (2013). Kinetics and competitive modelling of caesium biosortion onto chemically modified pine cone powder. J. Taiwan Inst. Chem. Eng..

[13] Wang J., Zhuang S. (2020). Caesium separation from radioactive waste by extraction and adsorption based on crown ethers and calixarenes. Nucl. Eng. Technol..

[14] Vaucher S., Li M., Mann S. (2000). Synthesis of prussian blue nanoparticles and nanocrystal superlattices in reverse microemulsions. Angew. Chem. Int. Ed..

[15] Yang H.M., Jang S.C., Hong S.B., Lee K.W., Roh C.H., Huh Y.S., Seo B.K. (2016). Prussian blue-functionalized magnetic nanoclusters for the removal of radioactive caesium from water. J. Alloys Compd..

[16] Grandjean F., Samain L., Long G.J. (2016). Characterization and utilization of prussian blue and its pigments. Dalton Trans..

[17] Samain L., Grandjean F., Long G.J., Martinetto P., Bordet P., Strivay D. (2013). Relationship between the synthesis of prussian blue pigments, their color, physical properties, and their behavior in paint layers. J. Phys. Chem. C.

[18] Kjeldgaard S., Dugulan I., Mamakhel A., Wagemaker M., Iversen B.B., Bentien A. (2021). Strategies for synthesis of prussian blue analogues. R. Soc. Open Sci..

[19] Oh D.M., Kim B.S., Kang S.W., Kim Y.S., Yoo S.J., Kim S., Chung Y.S., Choung S.W., Han J.H., Jung S.H. (2019). Enhanced immobilization of prussian blue through hydrogel formation by polymerization of acrylic acid for radioactive caesium adsorption. Sci. Rep..

[20] Takahashi A., Tanaka H., Minami K., Noda K., Ishizaki M., Kurihara M., Ogawac H., Kawamoto T. (2018). Unveiling Cs-adsorption mechanism of prussian blue analogs: Cs+-percolation via vacancies to complete dehydrated state. RSC Adv..

[21] Hong J.Y., Oh W.K., Shin K.Y., Kwon O.S., Son S., Jang J. (2012). Spatially controlled carbon sponge for targeting internalized radioactive materials in human body. Biomaterials.

[22] Nilchi A., Saberi R., Moradi M., Azizpour H., Zarghami R. (2011). Adsorption of caesium on copper hexacyanoferrate-PAN composite ion exchanger from aqueous solution. Chem. Eng. J..

[23] Sheha R.R. (2012). Synthesis and characterization of magnetic hexacyanoferrate (II) polymeric nanocomposite for separation of caesium from radioactive waste solutions. J. Colloid Interface Sci..

[24] Vincent C., Barré Y., Vincent T., Taulemesse J.M., Robitzer M., Guibal E. (2015). Chitin-prussian blue sponges for Cs(I) recovery: From synthesis to application in the treatment of accidental dumping of metal-bearing solutions. J. Hazard Mater..

[25] Vipin A.K., Fugetsu B., Sakata I., Isogai A., Endo M., Li M., Dresselhaus M.S. (2016). Cellulose nanofiber backboned Prus-sian blue nanoparticles as powerful adsorbents for the selective elimination of radioactive cesium. Sci. Rep..

[26] Kobayashi N., Yamamoto Y., Akashi M. (1998). Prussian blue as an agent for decontamination of ^137^Cs in radiation accidents. Jpn. J. Health Phys..

[27] Jang S.C., Hong S.B., Yang H.M., Lee K.W., Moon J.K., Seo B.K., Huh Y.S., Roh C. (2014). Removal of radioactive cesium using prussian blue magnetic nanoparticles. Nanomaterials.

[28] Gbewonyo S., Xiu S., Shahbazi A., Zhang L. (2020). Low thermal conductivity carbon material from electrospinning and subsequent chemical activation. Carbon Lett..

[29] Jung K.H., Kim S.J., Son Y.J., Ferraris J.P. (2019). Fabrication of carbon nanofiber electrodes using poly (acrylonitrile-co-vinylimidazole) and their energy storage performance. Carbon Lett..

[30] Khanlou H.M., Ang B.C., Talebian S., Afifi A.M., Andriyana A. (2014). Electrospinning of polymethyl methacrylate nanofibers: Optimization of processing parameters using the Taguchi design of experiments. Text. Res. J..

[31] Koysuren O., Koysuren H.N. (2016). Characterization of poly(methyl methacrylate) nanofiber mats by electrospinning process. J. Macromol. Sci. Part A.

[32] Lee J.J.L., Andriyana A., Ang B.C., Huneau B., Verron E. (2018). Electrospun PMMA polymer blend nanofibrous membrane: Electrospinability, surface morphology and mechanical response. Mater. Res. Express.

[33] Rameshkumar C., Sarojini S., Naresh K., Subalakshmi R. (2017). Preparation and characterization of pristine PMMA and PVDF thin film using solution casting process for optoelectronic devices. J. Surf. Sci. Technol..

[34] Vipin A.K., Hu B., Fugetsu B. (2013). Prussian blue caged in alginate/calcium beads as adsorbents for removal of caesium ions from contaminated water. J. Hazard Mater..

[35] Farah A.M., Billing C., Dikio C.W., Dibofori-Orji A.N., Oyedeji O.O., Wankasi D., Mtunzi F.M., Dikio E.D. (2013). Synthesis of prussian blue and its electrochemical detection of hydrogen peroxide based on cetyltrimethylammonium bromide (CTAB) modified glassy carbon electrode. Int. J. Electrochem. Sci..

[36] Aparicio C., Machala L., Marusak Z. (2012). Thermal decomposition of Prussian blue under inert atmosphere. J. Therm. Anal. Calorim..

[37] Langmuir I. (1918). The adsorption of gases on plane surfaces of glass, mica and platinum. J. Am. Chem. Soc..

[38] Freundlich H., Heller W. (1939). The adsorption of cis-and trans-azobenzene. J. Am. Chem. Soc..

[39] Rafatullah M., Sulaiman O., Hashim R., Ahmad A. (2009). Adsorption of copper (II), chromium (III), nickel (II) and lead (II) ions from aqueous solutions by meranti sawdust. J. Hazard Mater..

[40] Ho Y.S., Wase D.J., Forster C.F. (1996). Kinetic studies of competitive heavy metal adsorption by sphagnum moss peat. Environ. Technol..

[41] Mckay G., Bino M.J., Altamemi A.R. (1985). The adsorption of various pollutants from aqueous solutions on to activated carbon. Water Res..

